# Design and evaluation of antisense sequence length for modified mouse U7 small nuclear RNA to induce efficient pre-messenger RNA splicing modulation *in vitro*

**DOI:** 10.1371/journal.pone.0305012

**Published:** 2024-07-09

**Authors:** Takenori Shimo, Otoya Ueda, Satoshi Yamamoto

**Affiliations:** Research Division, Chugai Pharmaceutical Co., Ltd., Yokohama, Kanagawa, Japan; Florida Atlantic University, UNITED STATES

## Abstract

Pre-messenger RNA (pre-mRNA) splicing modulation is an attractive approach for investigating the mechanisms of genetic disorders caused by mis-splicing. Previous reports have indicated that a modified U7 small nuclear RNA (U7 snRNA) is a prospective tool for modulating splicing both *in vitro* and *in vivo*. To date, very few studies have investigated the role of antisense sequence length in modified U7 snRNA. In this study, we designed a series of antisense sequences with various lengths and evaluated their efficiency in inducing splicing modulation. To express modified U7 snRNAs, we constructed a series of plasmid DNA sequences which codes cytomegalovirus (CMV) enhancer, human U1 promoter, and modified mouse U7 snRNAs with antisense sequences of different lengths. We evaluated *in vitro* splicing modulation efficiency using a luciferase reporter system for simple and precise evaluation as well as reverse transcription-polymerase chain reaction to monitor splicing patterns. Our *in vitro* assay findings suggest that antisense sequences of modified mouse U7 snRNAs have an optimal length for efficient splicing modulation, which depends on the target exon. In addition, antisense sequences that were either too long or too short decreased splicing modulation efficiency. To confirm reproducibility, we performed an *in vitro* assay using two target genes, mouse *Fas* and mouse *Dmd*. Together, our data suggests that the antisense sequence length should be optimized for modified mouse U7 snRNAs to induce efficient splicing modulation.

## Introduction

Alternative pre-messenger RNA (mRNA) splicing is an essential process by which eukaryotes produce various proteins from limited numbers of genes. Previous studies have reported that mis-splicing triggers genetic disorders [1]. Therefore, splicing modulation is a potential tool for investigating the mechanisms of genetic disorders. In general, there are three approaches to splicing modulation: small molecule [2], antisense oligonucleotide (ASO) [3], and modified small nuclear RNAs (snRNAs) [4].

U7 snRNA is a U snRNA composed of U-rich ribonucleoproteins that induce the 3′ end processing of histone pre-mRNAs. Modified U7 snRNA-based splicing modulation was first described by *Grimm C*. *et al*. in 1993 [5]. For modified U7snRNAs, the Sm binding sequence was well optimized as a SmOpt sequence that inactivates the original function of U7 snRNA for processing histone pre-mRNA. To date, many studies have reported that splicing modulation can be induced by modified U7 snRNAs both *in vitro* and *in vivo* [4, 6–17]. However, few reports have investigated the effect of the length of the antisense sequence [18]. This may be important, as previous reports on ASOs revealed that selecting the proper ASO length is the key to enhancing splicing modulation efficiency [19–22]. In fact, LNA-modified ASOs are known to have an optimal length for effective splicing modulation [21]. Because a mechanism of splicing modulation by ASO is a steric blocking of splicing factors against pre-mRNA [23], longer ASOs form a highly stable duplex with the targeted pre-mRNA to achieve better splicing modulation efficiency. However, if the ASOs are too long, they inhibit splicing modulation efficiency because longer ASOs easily form secondary structures that prevent them from binding to the target pre-mRNA [24]. In this report, we hypothesized that the antisense sequence of modified U7 snRNA would also have an optimal length for efficient splicing modulation, which depends on the target exon.

In general, ASOs often use modified nucleic acids, such as 2’-*O*-methoxyethyl RNA (2’-MOE), phosphorodiamidate morpholino oligomers (PMO), 2’-*O*-methyl RNA (2’-OMe), 2’-*O*-4’-*C*-methylene-bridged nucleic acid (LNA/2’,4’-BNA). These chemical modifications enhance the binding stability between ASOs and target RNAs. Therefore, several previous reports have suggested that an appropriate length for chemically modified ASOs is 13-30-nt [19–21]. Conversely, modified U7 snRNAs consist of only native RNA because they are biologically synthesized with expression systems that utilize plasmid DNAs or virus vectors. Thus, compared to ASOs, we estimated that a much longer antisense sequence would be necessary for modified U7 snRNAs to obtain duplex stability against target RNAs. To date, antisense sequences of modified U7 snRNA normally have a length of 20-45-nt, though some exceptions exist [4, 6–17]. Therefore, in this report, we designed and evaluated long and short antisense sequences (9-149-nt) to determine the optimal length, which depends on the target exon, for modified U7 sRNAs to achieve efficient mRNA splicing modulation. Our results indicate that antisense sequence length affects the efficiency of splicing modulation.

## Material and methods

### Establishment of Luciferase reporter system

**Fig 1:**
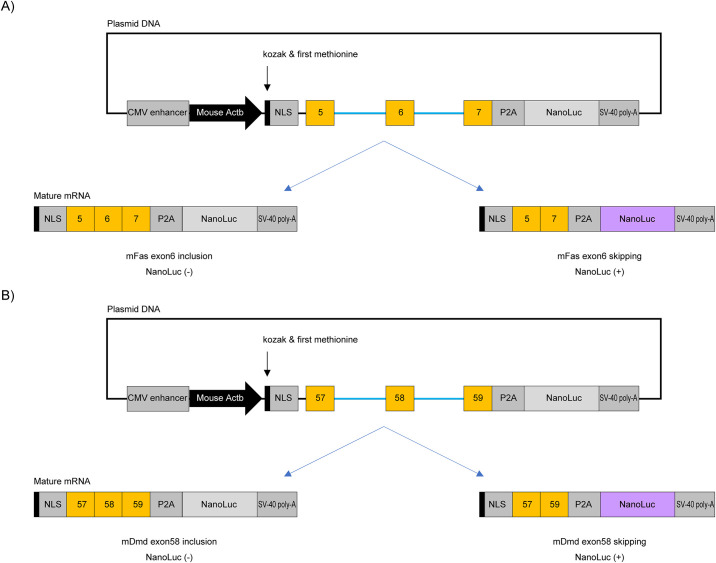
Schematic representation of Luciferase assay system for detecting splicing modulation of mouse *Fas* exon 6 and mouse *Dmd* exon 58 used in this study. Both exons and introns are indicated by orange box and narrow blue lines respectively. Mouse Actb: promoter sequence from mouse *actin-beta* (*Actb*) gene. NLS: nuclear localization signal. P2A: P2A sequence. NanoLuc: NanoLuc coding sequence. SV-40 poly-A: poly-A sequence from SV-40. A) When modified mouse U7 snRNA induces mouse *Fas* exon 6 skipping, a frame-shift triggers the expression of Luciferase protein. B) When modified mouse U7 snRNA induces mouse *Dmd* exon 58 skipping, a frame-shift triggers the expression of Luciferase protein.

We constructed plasmid DNAs for the Luciferase reporter system according to previous reports [21]. Minigene reporter plasmid DNAs coding either mouse *Fas* exon 6 or mouse *Dmd* exon 58 region were used for Luciferase reporter assays to evaluate splicing modulation efficiency according to previous report [25, 26]. Briefly, minigenes were amplified with mouse genome DNA from a C57BL/6J Jcl mouse. Because mouse *Dmd* intron 57 has a length of about 35 kbp, we removed the sequence of intron 57 from position +301 to +34922. Other introns used for the reporter system keep their original lengths. The NanoLuc coding sequences were obtained from a pNL1.1[NLuc] vector (Promega, Madison, WI). As a targeted exon was skipped, the frameshift mutation of pre-mRNA induces the expression of NanoLuc protein (Fig 1).

### Establishment of modified U7 snRNA expression system

We constructed plasmid DNAs which express the modified U7 snRNAs according to previous reports [11]. A mouse U7 snRNA sequence (GenBank accession no. X54165.1) was amplified from mouse genome DNA of C57BL/6J Jcl mouse. According to a previous report, endogenous expression of U7 snRNA is low (approximately 2–15×10^3), but converting the Sm binding sequence (AAUUUGUCUAG) into SmOpt sequence (AAUUUUUGGAG) enables accumulation in the nucleus and increases splicing modulation efficiency [5, 27, 28]. Therefore, we used SmOpt sequences in all expression systems in this study. Additionally, a previous report revealed that both human U1 snRNA promoter and terminal sequence increased the splicing modulation efficiencies of modified U7 snRNA [27]. Therefore, we applied the human U1 snRNA promoter for plasmid DNA expressing modified U7 snRNA in this report. Furthermore, a CMV enhancer was also used. Thus, we designed the series of U7 snRNA expression plasmid DNAs with both human U1 snRNA promoters and CMV enhancers (S1 Fig).

### Cell culture

In this study, we sought to evaluate modified mouse U7 snRNA for splicing modulation in mouse cell lines. Therefore, we used B16F10 cells originating from the C57BL/6J line for the assays [29]. B16F10 cells (TKG 0348, Cell Resource Center for Biomedical Research, Institute of Development, Aging and Cancer, Tohoku University) were cultured in Dulbecco’s modified Eagle Medium (DMEM) (Nacalai Tesque, Kyoto, Japan) containing 10% fetal bovine serum (FBS) (Citiva, Logan, UT or Sigma-Aldrich, St. Louis, MO), 1x penicillin streptomycin solution (Nacalai Tesque, Kyoto, Japan), and maintained in a 5% CO2 incubator at 37°C.

### RT-PCR analysis for the endogenous mouse *Fas* gene

For plasmid DNA transfection, B16F10 cells were seeded one day before transfection at a density of 100,000 cells in a 24-well plate (Corning, Corning, NY) and grown in DMEM (Nacalai Tesque) containing 10% FBS and 1x penicillin streptomycin solution. After 24 h, the cells were transfected with 1,000 ng of plasmid DNA expressing modified mouse U7 snRNA using Lipofectamine 3000 (Thermo Fisher Scientific, Waltham, MA) and Opti-MEM I (Thermo Fisher Scientific), according to the manufacturer’s protocols, and grown in DMEM containing 10% FBS and 1x penicillin streptomycin.

To collect total RNA from cells, the RNeasy Mini Kit (Qiagen, Hilden, Germany) was used. cDNA was prepared using ReverTraAce qPCR Mastermix with gDNA Remover (Toyobo, Osaka, Japan). A 10 μL volume of first-strand cDNA was synthesized from 500 ng of total RNA.

The cDNA was two-fold diluted with nuclease-free water (Toyobo). The cDNA was used as a template for individual PCR using specific primers (S1 Table). PCR reaction was performed using PrimeSTAR GXL DNA Polymerase (Takara Bio, Shiga, Japan). The PCR products were analyzed on a 2% agarose gel stained with GelRed (Biotium, Fremont, CA). Mouse *actin-beta* (*Actb*) was used as an internal control. DNA oligonucleotides were synthesized and purified by Eurofin Genomics K.K. (Tokyo, Japan). The intensity of each band was semi-quantified by using ImageJ software (1.47v, National Institutes of Health; from http://rsb.info.nih.gov/ij/). We encircled all the bands that underwent mouse *Fas* exon 6 inclusion (expected to be 345-bp) and mouse *Fas* exon 6 skipping (expected to be 291-bp) as rectangles of the same area. We then obtained the mean values using the measure mode. Each mean value was corrected by subtracting the mean value obtained from the water lane on the same gel, which was used as the background. The percentage of exon skipping was calculated by multiplying the mean value of exon skipped transcript by 100, relative to the total mean values of the exon skipped and exon included transcripts [25].

### Luciferase assay

B16F10 cells were seeded one day before transfection at a density of 1,500,000 cells in a T-75 flask (Corning) and grown in DMEM containing 10% FBS and 1x penicillin streptomycin solution. After 24 h, the cells were transfected with 400 ng (mouse *Fas* minigene) or 2,000 ng (mouse *Dmd* minigene) of luciferase reporter plasmid DNAs using Lipofectamine 3000 and Opti-MEM I, respectively, according to the manufacturer’s protocols, and grown in DMEM containing 10% FBS and 1x penicillin streptomycin solution. One hour after transfection, cells were suspended in 30 mL DMEM containing 10% FBS and 1x penicillin streptomycin solution and then re-seeded at 100 μL/well on a Nunc MicroWell 96-well, Nunclon Delta-Treated, Flat-Bottom Microplate (Thermo Fisher Scientific). Thirty minutes after re-seeding, 100 ng of modified mouse U7 snRNA expression plasmid DNAs was transfected with Lipofectamine 3000 and Opti-MEM I, according to the manufacturer’s protocols, and grown in DMEM containing 10% FBS and 1x penicillin streptomycin.

At 24 h after Luciferase reporter plasmid DNA transfection, to investigate luciferase activities, we used the NanoGlo Luciferase Assay System (Promega) reagent [30]. A mixture was prepared by combining the Nano-Glo Luciferase Assay Substrate with a 50-fold volume of the Nano-Glo Luciferase Assay Buffer. The mixture was then added at a volume of 50 μL/well and incubated for 5 minutes. The luciferase activities were detected with an Envision Multilabel Plate Reader (PerkinElmer, Waltham, MA). If modified U7snRNA induces splicing modulation (exon skipping), the frameshift triggers the expression of NanoLuc luciferase (Fig 1).

### RT-PCR analysis for the reporter mouse *Dmd* gene

B16F10 cells were seeded one day before transfection at a density of 1,500,000 cells in a T-75 flask (Corning) and grown in DMEM containing 10% FBS and 1x penicillin streptomycin solution. After 24 h, the cells were transfected with 2,000 ng (mouse *Dmd* minigene) of luciferase reporter plasmid DNAs using Lipofectamine 3000 and Opti-MEM I, respectively, according to the manufacturer’s protocols, and grown in DMEM containing 10% FBS and 1x penicillin streptomycin solution. One hour after transfection, cells were suspended in 30 mL DMEM containing 10% FBS and 1x penicillin streptomycin solution and then re-seeded at 500 μL/well on a 24-well plate (Corning). Thirty minutes after re-seeding, 500 ng of modified mouse U7 snRNA expression plasmid DNAs was transfected with Lipofectamine 3000 and Opti-MEM I, according to the manufacturer’s protocols, and grown in DMEM containing 10% FBS and 1x penicillin streptomycin.

At 24 h after Luciferase reporter plasmid DNA transfection, to collect total RNA from cells, the RNeasy Plus Mini Kit (Qiagen) was used. cDNA was prepared using ReverTraAce qPCR Mastermix with gDNA Remover (Toyobo). A 10 μL volume of first-strand cDNA was synthesized from 250 ng of total RNA.

The cDNA was two-fold diluted with nuclease-free water (Toyobo). The cDNA was used as a template for individual PCR using specific primers (S2 Table). PCR reaction was performed using PrimeSTAR GXL DNA Polymerase (Takara Bio). The PCR products were analyzed on a 1% or 2% agarose gel stained with Ethidium bromide (Nippon Gene, Toyama, Japan), GelRed (Biotium). Mouse *actin-beta* (*Actb*) was used as an internal control. DNA oligonucleotides were synthesized and purified by Eurofin Genomics K.K.

The intensity of each band was semi-quantified by using ImageJ software (1.47v, National Institutes of Health; from http://rsb.info.nih.gov/ij/). We encircled all the bands that underwent mouse *Dmd* exon 58 inclusion (expected to be 344-bp) and mouse *Dmd* exon 58 skipping (expected to be 223-bp) as rectangles of the same area. We then obtained the mean values using the measure mode. Each mean value was corrected by subtracting the mean value obtained from the water lane on the same gel, which was used as the background. The percentage of exon skipping was calculated by multiplying the mean value of exon skipped transcript by 100, relative to the total mean values of the exon skipped and exon included transcripts [25].

### Predicting melting temperature

We predicted the melting temperature (*T*_m_) for the antisense sequence of modified U7 snRNA against targeted mRNAs using the Biopython [31]. We used the Bio.SeqUtils.MeltingTemp module with the implemented RNA_NN3 table.

### Predicting secondary structures

To predict secondary structures and the minimum free energy (MFE; kcal/mol) for modified U7 snRNA, RNAfold algorithms of the ViennaRNA packages version 2.5.1 were used [32]. In the prediction, “A” was added to the beginning of antisense sequence as a transcription start site. Furthermore, the SmOpt sequence, “AAUUUUUGGAG”, and the mouse U7 snRNA terminal sequence, “CAGGUUUUCUGACUUCGGUCGGAAAACCCCU”, were added to the end of the antisense sequence as transcription termination sequences.

### Statistical analysis

Statistical analyses were performed with the software RStudio version 1.0.153 combined with R version 4.2.3. The Dwass-Steele-Critchlow-Fligner (DSCF) test was performed (dscfAllPairsTest function from the package PMCMRplus).

## Result

### Design of modified U7 snRNA targeting mouse *Fas* exon 6 and evaluation using both endogenous mouse *Fas* transcription and the luciferase reporter system

**Fig 2:**
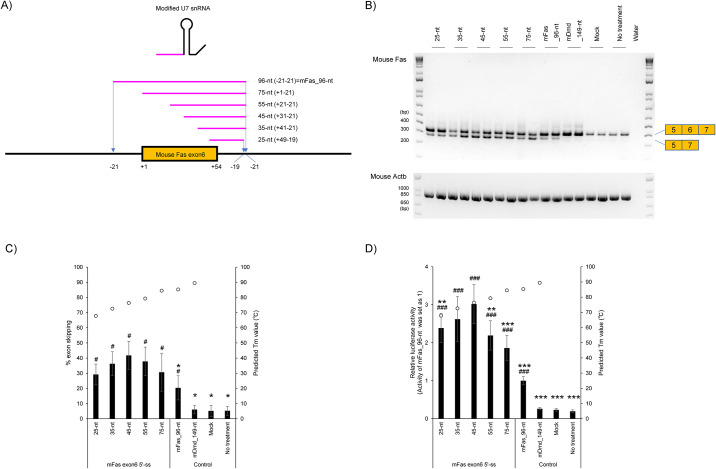
Evaluation of antisense sequences against mouse *Fas* exon 6. A) Schematic representation of designed antisense sequences against mouse *Fas* exon 6 for the assay. The notation in parentheses represents the target region of the antisense sequences. B) RT-PCR analyses show a full-length upper band (345-bp, mouse *Fas* exon 6 inclusion) and an exon skipped lower band (291-bp, mouse *Fas* exon 6 skipping). Mouse *Actb* was used as an internal control. Reproducible results were obtained from four independent experiments. Mock: treated with Lipofectamine only; no treatment: no transfection. C) The levels of endogenous mouse *Fas* exon 6 skipped mRNA in Fig 2B were semi-quantified. According to previous reports, the percentage exon skipping (% exon skipping) was calculated as the amount of exon skipped transcript relative to the total amount of exon skipped and full-length transcripts [25]. The graph shows the % exon skipping. Values represent the mean ± standard deviation of eight samples from four independent experiments performed in duplicate (n = 4, duplicate). Each open circle indicates a *T*_m_ value against RNA strand as predicted by Biopython. Mock: treated with Lipofectamine only; no treatment: no transfection. #*p* < 0.05 vs. Mock. *p < 0.05 vs. 45-nt. D)Result of Luciferase reporter assay and *T*_m_ prediction. Luciferase activity was detected to investigate the splicing modulation efficiency of antisense sequence targeting 5′ ss on mouse *Fas* exon 6. The graph shows that the normalized luciferase activity, relative to the value of the 96-nt antisense sequence. Values represent the mean ± standard deviation of eighteen samples from three independent experiments performed in sextuplicate (n = 3, sextuplicate). Each open circle indicates a predicted *T*_m_ value against an RNA strand by Biopython. Mock: treated with Lipofectamine only; no treatment: no transfection. ###*p* < 0.001 vs. Mock. **p < 0.01, ***p < 0.001 vs. 45-nt.

According to previous reports, designing an ASO to have an appropriate length is the key to enhancing splicing modulation efficiency [21, 22]. In fact, previous reports suggest that high *T*_m_ values contribute to the effectiveness of splicing modulation [19–21]. Therefore, we designed a series of modified U7 snRNAs targeting mouse *Fas* exon 6 that contains 25 to 96-nt long antisense sequences (Fig 2A and S3 Table). The designed antisense sequences spanned the 5′ splice site (ss) of mouse *Fas* exon 6 and parts of adjacent introns. Although previous studies on modified U7 snRNAs normally utilized 20-nt to 45-nt [4, 6–17], we also designed longer antisense sequences (≥45-nt) of modified U7 snRNAs because they are expected to have high *T*_m_ values. We then predicted the *T*_m_ values of each antisense sequence using biopython. The predicted *T*_m_ values are shown in the plots of Fig 2C and S3 Table. As we expected, the *T*_m_ values prediction by biopython showed that long antisense sequences increased *T*_m_ values.

We constructed a series of plasmid DNAs that express the modified U7 snRNAs containing designed antisense sequences. Plasmid DNAs were transfected into B16F10 cells and the splicing modulation efficiencies of designed modified U7 snRNA antisense sequences were evaluated (Fig 2B and 2C). We performed the RT-PCR analysis to detect the expression of the endogenous mouse *Fas* gene. The RT-PCR analysis showed that all designed 25-96-nt antisense sequences induced the splicing modulation efficiency (vs. Mock, *p* < 0.05). Among all designed antisense sequences, only the 96-nt antisense sequence targeting the whole mouse *Fas* exon 6 and adjacent intron regions showed the lower splicing modulation efficiency than the 45-nt antisense sequence in the assay (vs. 45-nt, *p* < 0.05, excepting the control).

Next, we constructed a luciferase reporter system for the brief and precise evaluation of mouse *Fas* exon skipping according to previous reports [25, 26] (Fig 1). With the reporter system, we also evaluated the 25 to 96-nt long antisense sequences (Fig 2D). The result showed that the 25-96-nt antisense sequences induced the splicing modulation efficiency (vs. Mock, *p* < 0.001). Additionally, if compared to 45-nt antisense sequence, only the 35-nt antisense sequence shows no significant difference. In details, the longer antisense sequences (≥55-nt) decreased splicing modulation efficiency in accordance with their length (vs. 45-nt, at least *p* < 0.01). The shorter antisense sequences (25-nt) decreased the splicing modulation efficiency as well (vs. 45-nt, *p* < 0.01). The 96-nt antisense sequence targeting the whole mouse *Fas* exon 6 and adjacent intron regions was the least active of the antisense sequences in this assay (25-75-nt antisense sequences vs. 96-nt, *p* < 0.001, excepting the control). The predicted *T*_m_ value of the 96-nt antisense sequence was 85.3°C, but that of 45-nt antisense sequence with the highest splicing modulation efficiency was 76.4°C (Fig 2 and S3 Table). Thus, we revealed that longer antisense sequences are not always better for inducing splicing modulation even though they increased the predicted *T*_m_ values against an RNA strand. Overall, our RT-PCR analysis and Luciferase reporter assays showed that antisense sequences of modified U7 snRNA, like those of ASOs, also have an optimal length.

### Design and evaluation of modified U7 snRNA antisense sequence targeting 5′ ss of mouse *Dmd* exon 58

**Fig 3:**
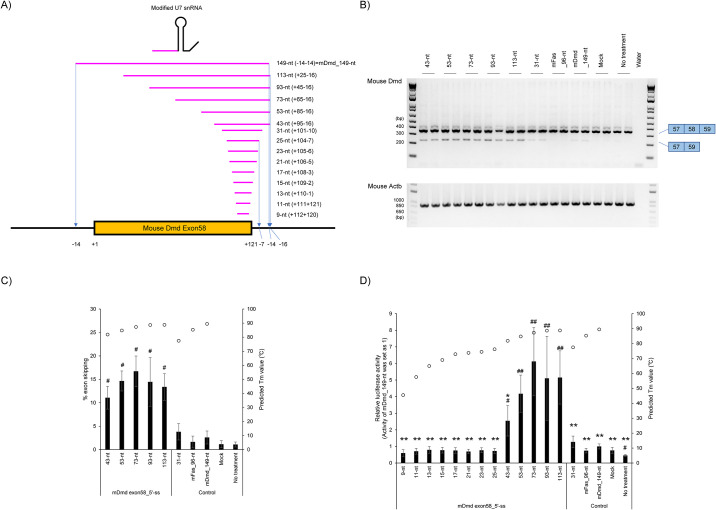
Evaluation of antisense sequences against mouse *Dmd* exon 58 targeting the 5′ ss site of mouse *Dmd* exon 58. A) Schematic representation of designed antisense sequences against mouse *Dmd* exon 58 for the assay. The notation in parentheses represents the target region of the antisense sequences. B) RT-PCR analyses show a full-length upper band (344-bp, mouse *Dmd* exon 58 inclusion) and an exon skipped lower band (223-bp, mouse *Dmd* exon 58 skipping). Mouse *Actb* was used as an internal control. Reproducible results were obtained from four independent experiments. Mock: treated with Lipofectamine only; no treatment: no transfection. C) The levels of endogenous mouse *Dmd* exon 58 skipped mRNA in Fig 3B were semi-quantified. According to previous reports, the percentage exon skipping (% exon skipping) was calculated as the amount of exon skipped transcript relative to the total amount of exon skipped and full-length transcripts [25]. The graph shows the % exon skipping. Values represent the mean ± standard deviation of eight samples from four independent experiments performed in duplicate (n = 4, duplicate). Each open circle indicates a predicted *T*_m_ value against RNA strand as predicted by Biopython. Mock: treated with Lipofectamine only; no treatment: no transfection. #*p* < 0.05 vs. Mock. D) Result of Luciferase reporter assay and *T*_m_ prediction. Luciferase activity was detected to investigate the splicing modulation efficiency of antisense sequence targeting 3′ ss on mouse *Dmd* exon 58. The graph shows the normalized luciferase activity, relative to the value of the 149-nt antisense sequence. Values represent the mean ± standard deviation (SD) of twelve samples from four independent experiments, each performed in triplicate (n = 4, triplicate). The plot shows that the predicted *T*_m_ values against RNA strands by the Biopython. Mock: treated with Lipofectamine only; no treatment: no transfection. #*p* < 0.05, ##*p* < 0.01 vs. Mock. *p < 0.05, **p < 0.01 vs. 73-nt.

In Fig 2, we revealed that the modified U7 snRNAs have an appropriate length for efficient splicing modulation of mouse *Fas* exon 6. To confirm the reproducibility with another gene, we constructed a luciferase reporter system which can monitor the splicing modulation efficiency of mouse *Dmd* exon 58 skipping. We designed 31-113-nt antisense sequences (Fig 3A and S4 Table). Each antisense sequence spans 5′ ss of mouse *Dmd* exon 58 and their adjacent introns. We also designed a 149-nt antisense sequence against the whole mouse *Dmd* exon 58 region and adjacent introns (Fig 3A and S4 Table). Plasmid DNAs were transfected into B16F10 cells which had been pre-transfected with luciferase reporter plasmid DNA, and the splicing modulation efficiencies of designed modified U7 snRNA antisense sequences were evaluated (Fig 3B–3D).

We performed the RT-PCR analysis to detect the expression of the reporter mouse *Dmd* mini-gene (Fig 3B and 3C). The RT-PCR analysis showed that the 43-113-nt antisense sequences induced splicing modulation efficiency (vs. Mock, *p* < 0.05). In contrast, shorter or longer antisense sequences, such as 31-nt and 149-nt antisense sequences showed no significant splicing modulation efficiency (vs. Mock, not significant). Next, we performed the luciferase reporter assay. As a result, 43-113-nt antisense sequences induced the splicing modulation efficiency (vs. Mock, at least *p* < 0.05). In contrast, the 149-nt antisense sequence against whole mouse *Dmd* exon 58 and adjacent introns could not induce enough splicing modulation efficiency (vs. Mock, not significant). If compared to a 73-nt antisense sequence, 53-nt, 93-nt, and 113-nt antisense sequences showed similar splicing modulation efficiency (no statistic significant). On the other hand, both shorter (≤43-nt) and longer (149-nt) antisense sequences showed a low splicing modulation efficiency (vs. 73-nt, at least *p* < 0.05).

The predicted *T*_m_ values by biopython are shown in the graph in Fig 3 and S4 Table. The predicted *T*_m_ value of 73-nt antisense sequence which showed high splicing efficiency was 87.3°C. In contrast, the predicted *T*_m_ value of 149-nt antisense sequence was 89.5°C. Thus, although 149-nt showed a higher predicted *T*_m_ value than that of 73-nt, the 149-nt antisense sequence could not induce effective splicing modulation.

In previous studies regarding modified U7snRNA, antisense sequences of modified U7 snRNA normally have a length of 20-45-nt, but ASOs are normally 13-30-nt. Therefore, we sought to design various short antisense sequences targeting 5′ ss of mouse *Dmd* exon 58 (9-25-nt) (Fig 3A and S4 Table) to determine whether a short antisense sequence of modified U7 snRNA induces efficient splicing modulation. The luciferase reporter assay revealed that each short antisense sequence did not show splicing modulation (vs. Mock, not significant) (Fig 3D). The predicted *T*_m_ values are shown in the graph of Fig 3D and S4 Table. The predicted *T*_m_ values of short antisense sequences ranged from 45.5 to 76.2°C.

In summary, our luciferase reporter assays in Fig 3 confirmed the reproducibility of the result in Fig 2. Thus, longer modified U7 snRNA antisense sequences, such as 149-nt, are not always better for inducing splicing modulation, and antisense sequences targeting the *Dmd* gene, like ASOs, also have an optimal length. Additionally, the result in Figs 2 and 3 shows that the appropriate length differs depending on the targeted exons. Moreover, much shorter antisense sequences (9-25-nt) of modified U7 snRNA could not induce efficient splicing modulation.

### Design and evaluation of modified U7 snRNA antisense sequence targeting 3′ ss of mouse *Dmd* exon 58

**Fig 4:**
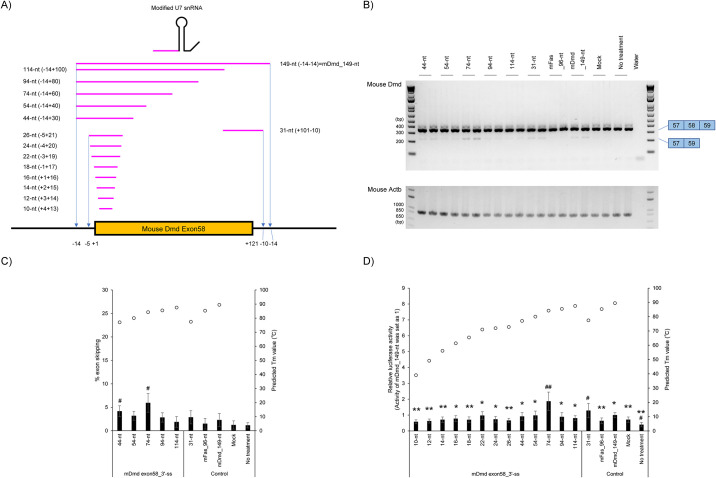
Evaluation of antisense sequences against mouse *Dmd* exon 58 targeting the 3′ ss site of mouse *Dmd* exon 58. A) Schematic representation of designed antisense sequences against mouse *Dmd* exon 58 for the assay. The notation in parentheses represents the target region of the antisense sequences. B) RT-PCR analyses show a full-length upper band (344-bp, mouse *Dmd* exon 58 inclusion) and an exon skipped lower band (223-bp, mouse *Dmd* exon 58 skipping). Mouse *Actb* was used as an internal control. Reproducible results were obtained from four independent experiments. Mock: treated with Lipofectamine only; no treatment: no transfection. C) The levels of endogenous mouse *Dmd* exon 58 skipped mRNA in Fig 3B were semi-quantified. According to previous reports, the percentage exon skipping (% exon skipping) was calculated as the amount of exon skipped transcript relative to the total amount of exon skipped and full-length transcripts [25]. The graph shows the % exon skipping. Values represent the mean ± standard deviation of eight samples from four independent experiments performed in duplicate (n = 4, duplicate). Each open circle indicates a predicted *T*_m_ value against RNA strand as predicted by Biopython. Mock: treated with Lipofectamine only; no treatment: no transfection. #*p* < 0.05 vs. Mock. D) Result of Luciferase reporter assay and *T*_m_ prediction. Luciferase activity was detected to investigate the splicing modulation efficiency of antisense sequences targeting 3′ ss on mouse *Dmd* exon 58. The graph shows that the normalized luciferase activity, relative to the value of 149-nt antisense sequence. Values represent the mean ± standard deviation (SD) of twelve samples from four independent experiments, each performed in triplicate (n = 4, triplicate). Each open circle indicates a predicted *T*_m_ value against an RNA strand by Biopython. Mock: treated with Lipofectamine only; no treatment: no transfection. #*p* <0.05, ##*p* < 0.01 vs. Mock. *p < 0.05, ** *p* < 0.01 vs. 74-nt.

In Figs 2 and 3, we revealed that the modified U7 snRNAs have an appropriate length for efficient splicing modulation, which depends on the target exon. We also showed that shorter antisense sequences did not induce splicing modulation efficiency. To confirm the reproducibility, we also designed 10-114-nt antisense sequences (Fig 4A and S5 Table). Each designed antisense sequence spans 3′ ss of mouse *Dmd* exon 58 and their adjacent introns. Plasmid DNAs were transfected into B16F10 cells which had been pre-transfected with luciferase reporter plasmid DNA, and the splicing modulation efficiencies of designed modified U7 snRNA antisense sequences were evaluated (Fig 4B–4D).

We performed RT-PCR analysis to detect the expression of the reporter mouse *Dmd* minigene (Fig 4B and 4C). The RT-PCR analysis showed that only 44-nt and 74-nt antisense sequences enabled the slight induction of splicing modulation (vs. Mock, *p* < 0.05). In contrast, other antisense sequence showed no splicing modulation efficiency (vs. Mock, not significant).

Next, we also performed a luciferase reporter assay revealing that the designed antisense sequences have optimal lengths, but which depend on the target exon (Fig 4D). In more detail, both the 31-nt and 74-nt antisense sequences enabled the slight induction of splicing modulation (vs. Mock, at least *p* < 0.05). Conversely, shorter (≤54-nt) sequences, with the exception of a 31-nt antisense sequence, and longer (≥94-nt) antisense sequences decreased splicing modulation efficiency.

The predicted *T*_m_ values by biopython are shown in the graph in Fig 4 and S5 Table. The predicted *T*_m_ value of the 74-nt antisense sequence which showed a high splicing modulation efficiency was 84.2°C In contrast, the predicted *T*_m_ value of 149-nt antisense sequence was 89.5°C. Thus, although 149-nt showed a higher predicted *T*_m_ value than 74-nt, the 149-nt antisense sequence could not induce effective splicing modulation.

To summarize the results, Fig 4 confirmed the reproducibility of the result in Figs 2 and 3. In particular, short antisense sequences (10-26-nt) of modified U7 snRNA could not induce efficient splicing modulation compared to the longer antisense sequence (i.e. 74-nt).

## Discussion

Previous studies on ASO have stated that proper ASO design is a key to enhancing splicing modulation efficiency [19, 20]. For example, *Aartsma-Rus et al*. reported that the several ASO parameters, such as GC contents and *T*_m_ values against the target RNA, differ between effective and ineffective ASOs [19]. Other previous studies also revealed that both the optimal length and secondary structure of ASOs affect the splicing modulation efficiency [21]. Based on these ASO studies, we aimed to design the appropriate length of antisense sequence for modified U7 snRNA in this study. To enhance the *T*_m_ values of antisense sequences against target mRNA, we designed longer antisense sequences (45-149-nt). This is because, unlike ASOs that can be chemically modified to enhance the binding affinity against target mRNA, modified U7 snRNAs cannot be similarly chemically modified as they are biologically synthesized with expression systems using plasmid DNAs or virus vectors. In fact, our *in vitro* study revealed that both the short antisense sequences (9-26-nt) of modified U7 sRNAs could not induce efficient splicing modulation, even though ASOs normally have a length of less than 30-nt [33]. As we expected, the long antisense sequences, such as 35-45-nt against mouse *Fas* exon 6 and 53-113-nt against mouse *Dmd* exon 58, showed the highest splicing modulation efficiency among all the designed sequences. To date, antisense sequences of modified U7snRNA have had a length of 20-45-nt, but there are some exceptions [4, 6–17]. Thus, we recommend that longer antisense sequences should also be designed when initially screening modified U7 snRNAs using an *in vitro* assay or when predicting both the *T*_m_ value and secondary structure *in-silico*.

In a previous report on ASOs, a cocktail of multiple ASOs which tile against a whole human *DMD* exon 51 region efficiently induced exon skipping [25]. Therefore, we designed the antisense sequence against the whole exon and adjacent intron regions in this study (96-nt against mouse *Fas* exon6 and 149-nt against mouse *Dmd* exon58). However, our *in vitro* assay indicated that the longer antisense sequence did not enhance exon skipping. One possible explanation is that the secondary structure formed by the long antisense sequence inhibits splicing modulation efficiency. In fact, a previous ASO study reported that either inter- or inner- secondary structures of ASOs decrease the efficiency of splicing modulation [24]. To predict the formation of the secondary structure by the modified U7 snRNA antisense sequence, we used the RNAfold algorithms of the ViennaRNA packages as a secondary structure prediction program (S2–S4 Figs). As a result, the longer antisense sequences, such as 96-nt against mouse *Fas* exon 6 and 149-nt against mouse *Dmd* exon 58, are predicted to form more stable secondary structures than shorter antisense sequences (S2 and S3 Figs). In contrast, the shorter antisense sequences, such as 45-nt against mouse *Fas* exon 6, 73-nt against 5′ ss of mouse *Dmd* exon 58, and 74-nt against 3′ ss of mouse *Dmd* exon 58 are predicted to form unstable secondary structures (S2–S4 Figs). Thus, we suggest that, to optimize modified U7 snRNA antisense sequences, the formation of secondary structures must be avoided.

Splicing modulation using modified U7 snRNAs is a prospective strategy to artificially control a pre-mRNA splicing both *in vitro* and *in vivo*. In fact, many previous studies have reported that modified U7 snRNA enables the splicing modulation of various genes *in vivo*, such as in a Duchenne muscular dystrophy (DMD) mouse model (D2-mdx mice [4], mdx mice [9], mdx52 mice [16], utrophin/dystrophin double-knockout mice [13], *Dmd* exon 2 duplication model mice [34]), an amyotrophic lateral sclerosis (ALS) mouse model [35], and a spinal muscular atrophy (SMA) mouse model [36]. Although we did not perform *in vivo* assays in this study, our results suggest that having an antisense sequence of optimal length, which depends on the target exon, allows modified U7 snRNA to induce efficient splicing modulation *in vivo*. In future experiments, it is necessary to evaluate the use of *in vivo* expression systems using virus vectors, mRNAs, and transgenic insertions for synthesizing modified U7 snRNA.

*Suter et al*. previously suggested that one prospective strategy to enhance splicing modulation efficiencies is to use a modified U7snRNA containing double-target antisense sequences [18]. On the contrary, *Aupy et al*. reported that the 45-nt single-target antisense sequence demonstrated more effective splicing modulation than the double-target antisense sequence, which contained 20-nt and 23-nt sequences [16]. To date, double-target antisense sequences have targeted divided regions with two antisense sequences, each typically ranging from 16-28-nt in length [16, 18, 37]. Although we did not design the double-target antisense in this study, we think that the optimal length should also be investigated for double-target antisense sequences. Therefore, further studies are necessary to compare modified U7snRNA containing the optimized length of double-target antisense sequences with the optimized length of a single-target antisense sequence.

In conclusion, we designed a series of modified mouse U7 snRNA antisense sequences and evaluated their splicing modulation efficiency *in vitro*. As a result, we found that the antisense sequence of modified mouse U7snRNA has an optimal length for inducing efficient splicing modulation, which depends on the target exon. Additionally, we revealed that antisense sequences that were either too long or too short decreased splicing modulation efficiency.

## Supporting information

S1 Table DNA primers for RT-PCR of Fig 2. Sequences are shown from 5′ to 3′. Uppercase letters: DNA.
(PDF)

S2 Table DNA primers for RT-PCR of Figs 3 and 4. Sequences are shown from 5′ to 3′. Uppercase letters: DNA.
(PDF)

S3 Table Antisense sequences of U7 snRNA for Fig 2. Sequences are shown from 5′ to 3′. Lowercase letters: RNA. Predicted *T*_m_ value was predicted with Biopython. Predicted MFE (minimum free energy) was predicted with RNAfold algorithms of the ViennaRNA packages.
(PDF)

S4 Table Antisense sequences of U7 snRNA for Fig 3. Sequences are shown from 5′ to 3′. Lowercase letters: RNA. Predicted *T*_m_ value was predicted with Biopython. Predicted MFE (minimum free energy) was predicted with RNAfold algorithms of the ViennaRNA packages.
(PDF)

S5 Table Antisense sequences of U7 snRNA for Fig 4. Sequences are shown from 5′ to 3′. Lowercase letters: RNA. Predicted *T*_m_ value was predicted with Biopython. Predicted MFE (minimum free energy) was predicted with RNAfold algorithms of the ViennaRNA packages.
(PDF)

S1 Fig Schematic representation of plasmid DNA that express modified U7 snRNA for the assay. Schematic representation of plasmid DNA used for the assay. The modified mouse U7 snRNA is expressed by a human U1 snRNA promoter and a CMV enhancer. The modified mouse U7 snRNA contains antisense sequence targeting specific gene of interest, SmOpt sequence, and mouse U7 snRNA termination sequence.
(PDF)

S2 Fig Prediction of secondary structure formed by long antisense sequence on modified U7 snRNA. Reliability plot and minimum free energy (MFE) of antisense sequences on modified U7 snRNA targeting mouse *Fas* exon 6. A) 25-nt, B) 35-nt, C) 45-nt, D) 55-nt, E) 75-nt, and F) 96-nt respectively. ViennaRNA packages version 2.5.1 was used for prediction.
(PDF)

S3 Fig Prediction of secondary structure formed by long antisense sequence on modified U7 snRNA. Reliability plot and minimum free energy (MFE) of antisense sequences on modified U7 snRNA targeting mouse *Dmd* exon 58. A) 43-nt, B) 53-nt, C) 73-nt, D) 93-nt, E) 113-nt, F) 31-nt and G) 149-nt respectively. ViennaRNA packages version 2.5.1 was used for prediction.
(PDF)

S4 Fig Prediction of secondary structure formed by long antisense sequence on modified U7 snRNA. Reliability plot and minimum free energy (MFE) of antisense sequences on modified U7 snRNA targeting mouse *Dmd* exon 58. A) 44-nt, B) 54-nt, C) 74-nt, D) 94-nt and E) 114-nt respectively. ViennaRNA packages version 2.5.1 was used for prediction.
(PDF)

S1 Raw image A) The original gel for Fig 2. This gel indicated the result of RT-PCR analysis targeting mouse *Fas*. B) The original gel for Fig 2. This gel indicated the result of RT-PCR analysis targeting mouse *Actb*. C) The original gel for Fig 3. This gel indicated the result of RT-PCR analysis targeting mouse *Dmd* minigene. D) The original gel for Fig 3. This gel indicated the result of RT-PCR analysis targeting mouse *Actb*. E) The original gel for Fig 4. This gel indicated the result of RT-PCR analysis targeting mouse *Dmd* minigene. F) The original gel for Fig 4. This gel indicated the result of RT-PCR analysis targeting mouse *Actb*.
(PDF)
