# Supplementary material for: Design and evaluation of antisense sequence length for modified mouse U7 small nuclear RNA to induce efficient pre-messenger RNA splicing modulation in vitro
Source: PLoS One. 2024 Jul 9;19(7):e0305012. doi: 10.1371/journal.pone.0305012 (PMC11232981; doi:10.1371/journal.pone.0305012)
Supplement: S1 Table — Sequences are shown from 5′ to 3′. Uppercase letters: DNA. (PDF) [file pone.0305012.s001.pdf]

S1 Table. DNA primers for RT-PCR of Fig 2

	Entry	Sequence (5′--3′)	PCR product length
1	Forward primer	CAAGTGCAAACCAGACTTCTACTGCG	345 bp (mouse Fas exon6 inclusion)
2	Reverse primer	TTAGCTTCCTGGATTGTCATGTCTTCAG	291 bp (mouse Fas exon6 skipping)

	Entry	Sequence (5′--3′)	PCR product length
1	Forward primer	CCATGGATGACGATATCGCTGCGCTG	869 bp (mouse Actb)
2	Reverse primer	GATGTCAACGTCACACTTCATGATGGAATTG	

Sequences are shown from 5′ to 3′. Uppercase letters: DNA.
