# Supplementary material for: Design and evaluation of antisense sequence length for modified mouse U7 small nuclear RNA to induce efficient pre-messenger RNA splicing modulation in vitro
Source: PLoS One. 2024 Jul 9;19(7):e0305012. doi: 10.1371/journal.pone.0305012 (PMC11232981; doi:10.1371/journal.pone.0305012)
Supplement: S2 Table — Sequences are shown from 5′ to 3′. Uppercase letters: DNA. (PDF) [file pone.0305012.s002.pdf]

S2 Table. DNA primers for RT-PCR of Figs 3 and 4

Entry	Sequence (5′--3′)	PCR product length
1 Forward primer	GAAGCAAGTTCTGACCAGTGGAAGCG	344 bp (mouse Dmd exon58 inclusion)
2 Reverse primer	CATTCAGCGTTGACCTCTTCAGCCTG	223 bp (mouse Dmd exon58 skipping)

Entry	Sequence (5′--3′)	PCR product length
1 Forward primer	CCATGGATGACGATATCGCTGCGCTG	869 bp (mouse Actb)
2 Reverse primer	GATGTCAACGTCACACTTCATGATGGAATTG	

Sequences are shown from 5' to 3'. Uppercase letters: DNA.
