# Supplementary material for: Design and evaluation of antisense sequence length for modified mouse U7 small nuclear RNA to induce efficient pre-messenger RNA splicing modulation in vitro
Source: PLoS One. 2024 Jul 9;19(7):e0305012. doi: 10.1371/journal.pone.0305012 (PMC11232981; doi:10.1371/journal.pone.0305012)
Supplement: S3 Table — Sequences are shown from 5′ to 3′. Lowercase letters: RNA. Predicted Tm value was predicted with Biopython. Predicted MFE (minimum free energy) was predicted with RNAfold algorithms of the ViennaRNA packages. (PDF) [file pone.0305012.s003.pdf]

S3 Table. Antisense sequences of U7 snRNA for Fig 2

	Entry	Sequence (5′--3′)	GC%	Predicted T_m (°C)	Predicted MFE (kcal/mol)
1	mFas_5'-ss_25-nt	gaacaaacuuaggacuuaccaagug	40.0	67.9	-19.6
2	mFas_5'-ss_35-nt	uugaacaaacuuaggacuuaccaaguggaauuaac	34.3	72.6	-18.8
3	mFas_5'-ss_45-nt	uugaacaaacuuaggacuuaccaaguggaauuaacaaaacaagga	33.3	76.4	-19.4
4	mFas_5'-ss_55-nt	uugaacaaacuuaggacuuaccaaguggaauuaacaaaacaaggaugguc aacaa	34.5	79.3	-21.9
5	mFas_5'-ss_75-nt	uugaacaaacuuaggacuuaccaaguggaauuaacaaaacaaggaugguc aacaaccauaggcgauuucugggac	40.0	84.5	-25.5
6	full_mFas_exon6+ adjacent introns (mFas_96-nt)	uugaacaaacuuaggacuuaccaaguggaauuaacaaaacaaggaugguc aacaaccauaggcgauuucugggaccugcgaauauuggauauuuug	38.5	85.3	-30.9
7	full_mDmd_exon58 +adjacent introns (mDmd_149-nt)	ccacauucaauuaccucugggcuccugguagaguuucucuaguccuucc aaaggcugcucugucagaaauauucucacagucuccagaguacucauga uuacagguucuuuaguuuucaauucccucuugaaggccugugaaaugag au	43.0	89.5	-56.7

Sequences are shown from 5′ to 3′. Lowercase letters: RNA. Predicted T_m value was predicted with Biopython. Predicted MFE (minimum free energy) was predicted with RNAfold algorithms of the ViennaRNA packages.
