# Supplementary material for: Design and evaluation of antisense sequence length for modified mouse U7 small nuclear RNA to induce efficient pre-messenger RNA splicing modulation in vitro
Source: PLoS One. 2024 Jul 9;19(7):e0305012. doi: 10.1371/journal.pone.0305012 (PMC11232981; doi:10.1371/journal.pone.0305012)
Supplement: S4 Table — Sequences are shown from 5′ to 3′. Lowercase letters: RNA. Predicted Tm value was predicted with Biopython. Predicted MFE (minimum free energy) was predicted with RNAfold algorithms of the ViennaRNA packages. (PDF) [file pone.0305012.s004.pdf]

S4 Table. Antisense sequences of U7 snRNA for Fig 3

	Entry	Sequence (5'--3')	GC%	Predicted T_m (°C)	Predicted MFE (kcal/mol)
1	mDmd_5'-ss_9-nt	ucugggcuc	66.7	45.5	-21.2
2	mDmd_5'-ss_11-nt	cucugggcucc	72.7	57.6	-25.7
3	mDmd_5'-ss_13-nt	ccucugggcuccu	69.2	65.0	-25.2
4	mDmd_5'-ss_15-nt	accucugggcuccug	66.7	69.0	-24.3
5	mDmd_5'-ss_17-nt	uaccucugggcuccugg	64.7	72.8	-24.2
6	mDmd_5'-ss_21-nt	auuaccucugggcuccuggua	52.4	73.8	-24.0
7	mDmd_5'-ss_23-nt	aauuaccucugggcuccugguag	52.2	74.5	-23.9
8	mDmd_5'-ss_25-nt	caauuaccucugggcuccugguaga	52.0	76.2	-23.8
9	mDmd_5'-ss_43-nt	uuccacauucaauuaccucugggcuccugguagaguuucucua	44.2	81.8	-27.1
10	mDmd_5'-ss_53-nt	uuccacauucaauuaccucugggcuccugguagaguuucucuaguccuuccaa	45.3	84.7	-26.9
11	mDmd_5'-ss_73-nt	uuccacauucaauuaccucugggcuccugguagaguuucucuaguccuuccaaaggcugcucugucagaaaua	45.2	87.3	-34.1
12	mDmd_5'-ss_93-nt	uuccacauucaauuaccucugggcuccugguagaguuucucuaguccuuccaaaggcugcucugucagaaauauucucacagucuccagagua	45.2	88.7	-39.4
13	mDmd_5'-ss_113-nt	uuccacauucaauuaccucugggcuccugguagaguuucucuaguccuuccaaaggcugcucugucagaaauauucucacagucuccagaguacucaugauuacagguucuuu	43.4	88.8	-39.4

14	mDmd_5'-ss_31-nt	auucaauuaccucugggcuccugguagaguu	45.2	77.4	-23.6
15	full_mFas_exon6+ adjacent introns (mFas_96-nt)	uugaacaaacuuaggacuuaccaaguggaauuaacaaaacaaggauggu caacaaccauaggcgauuucugggaccugcgaauauuggauauuuug	38.5	85.3	-30.9
16	full_mDmd_exon58 +adjacent introns (mDmd_149-nt)	ccacauucaauuaccucugggcuccugguagaguuucucuaguccuucc aaaggcugcucugucagaaauauucucacagucuccagaguacucauga uuacagguucuuuaguuuucaauucccucuugaaggccugugaaaugag au	43.0	89.5	-56.7

Sequences are shown from 5′ to 3′. Lowercase letters: RNA. Predicted T_m value was predicted with Biopython. Predicted MFE (minimum free energy) was predicted with RNAfold algorithms of the ViennaRNA packages.
