# Supplementary material for: Design and evaluation of antisense sequence length for modified mouse U7 small nuclear RNA to induce efficient pre-messenger RNA splicing modulation in vitro
Source: PLoS One. 2024 Jul 9;19(7):e0305012. doi: 10.1371/journal.pone.0305012 (PMC11232981; doi:10.1371/journal.pone.0305012)
Supplement: S5 Table — Sequences are shown from 5′ to 3′. Lowercase letters: RNA. Predicted Tm value was predicted with Biopython. Predicted MFE (minimum free energy) was predicted with RNAfold algorithms of the ViennaRNA packages. (PDF) [file pone.0305012.s005.pdf]

S5 Table. Antisense sequences of U7 snRNA for Fig 4

	Entry	Sequence (5′--3′)	GC%	Predicted T_m (°C)	Predicted MFE (kcal/mol)
1	mDmd_3'-ss_10-nt	cccucuugaa	50.0	39.0	-17.7
2	mDmd_3'-ss_12-nt	ucccucuugaag	50.0	49.1	-18.5
3	mDmd_3'-ss_14-nt	uucccucuugaagg	50.0	56.1	-18.8
4	mDmd_3'-ss_16-nt	auucccucuugaaggc	50.0	61.4	-19.6
5	mDmd_3'-ss_18-nt	aauucccucuugaaggcc	50.0	65.4	-19.5
6	mDmd_3'-ss_22-nt	ucaauucccucuugaaggccug	50.0	71.0	-19.2
7	mDmd_3'-ss_24-nt	uucaauucccucuugaaggccugu	45.8	72.0	-19.7
8	mDmd_3'-ss_26-nt	uuucaauucccucuugaaggccugug	46.2	72.8	-19.4
9	mDmd_3'-ss_44-nt	uucuuuaguuuucaauucccucuugaaggccugugaaaugagau	36.4	77.1	-22.3
10	mDmd_3'-ss_54-nt	ugauuacagguucuuuaguuuucaauucccucuugaaggccugugaaaugagau	37.0	80.0	-27.8
11	mDmd_3'-ss_74-nt	cacagucuccagaguacucaugauuacagguucuuuaguuuucaauucccucuugaaggccugugaaaugagau	40.5	84.2	-41.5
12	mDmd_3'-ss_94-nt	ugcucugucagaaauauucucacagucuccagaguacucaugauuacagguucuuuaguuuucaauucccucuugaaggccugugaaaugagau	39.4	85.5	-46.2
13	mDmd_3'-ss_114-nt	ucucuaguccuuccaaaggcugcucugucagaaauauucucacagucuccagaguacucaugauuacagguucuuuaguuuucaauucccucuugaaggccugugaaaugagau	41.2	87.5	-49.5

14	mDmd_5'-ss_31-nt	auucaauuaccucugggcuccugguagaguu	45.2	77.4	-23.6
15	full_mFas_exon6+ adjacent introns (mFas_96-nt)	uugaacaaacuuaggacuuaccaaguggaauuaacaaaacaaggauggu caacaaccauaggcgauuucugggaccugcgaauauuggauauuuug	38.5	85.3	-30.9
16	full_mDmd_exon58 +adjacent introns (mDmd_149-nt)	ccacauucaauuaccucugggcuccugguagaguuucucuaguccuucc aaaggcugcucugucagaaauauucucacagucuccagaguacucauga uuacagguucuuuaguuuucaauucccucuugaaggccugugaaaugag au	43.0	89.5	-56.7

Sequences are shown from 5′ to 3′. Lowercase letters: RNA. Predicted T_m value was predicted with Biopython. Predicted MFE (minimum free energy) was predicted with RNAfold algorithms of the ViennaRNA packages.
