# Supplementary material for: Design and evaluation of antisense sequence length for modified mouse U7 small nuclear RNA to induce efficient pre-messenger RNA splicing modulation in vitro
Source: PLoS One. 2024 Jul 9;19(7):e0305012. doi: 10.1371/journal.pone.0305012 (PMC11232981; doi:10.1371/journal.pone.0305012)

S1 Fig. Schematic representation of plasmid DNA that express modified U7 snRNA for the assay.

Schematic representation of plasmid DNA used for the assay. The modified mouse U7 snRNA is expressed by a human U1 snRNA promoter and a CMV enhancer. The modified mouse U7 snRNA contains antisense sequence targeting specific gene of interest, SmOpt sequence, and mouse U7 snRNA termination sequence.

S1 Fig.

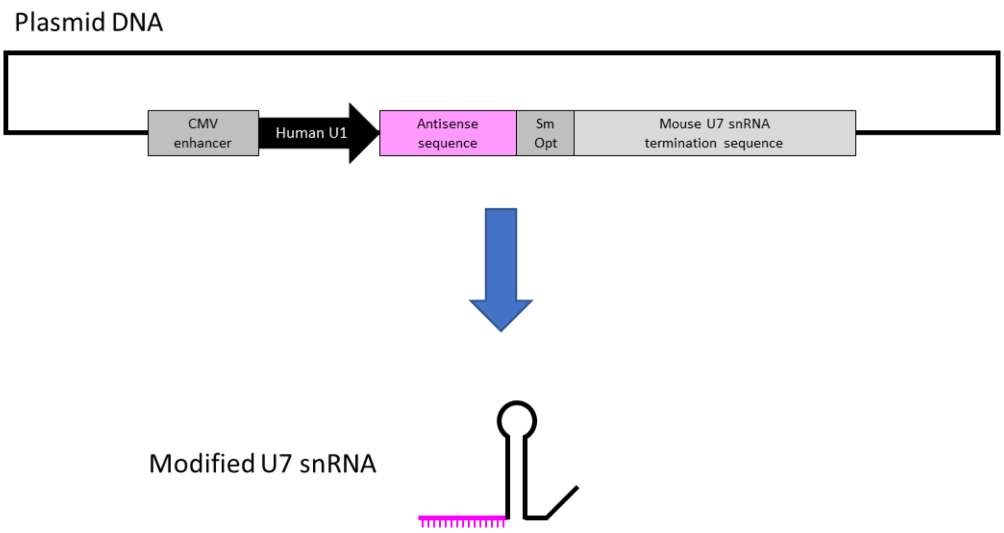

Supplement: S1 Fig — Schematic representation of plasmid DNA used for the assay. The modified mouse U7 snRNA is expressed by a human U1 snRNA promoter and a CMV enhancer. The modified mouse U7 snRNA contains antisense sequence targeting specific gene of interest, SmOpt sequence, and mouse U7 snRNA termination sequence. (PDF) [file pone.0305012.s006.pdf]
