# Supplementary material for: Design and evaluation of antisense sequence length for modified mouse U7 small nuclear RNA to induce efficient pre-messenger RNA splicing modulation in vitro
Source: PLoS One. 2024 Jul 9;19(7):e0305012. doi: 10.1371/journal.pone.0305012 (PMC11232981; doi:10.1371/journal.pone.0305012)

**S2 Fig. Prediction of secondary structure formed by long antisense sequences on modified U7 snRNA.**

Reliability plot and minimum free energy (MFE) of antisense sequences on modified U7 snRNA targeting mouse *Fas* exon 6. A) 25-nt, B) 35-nt, C) 45-nt, D) 55-nt, E) 75-nt, and F) 96-nt, respectively. ViennaRNA packages version 2.5.1 was used for prediction.

S2 Fig.

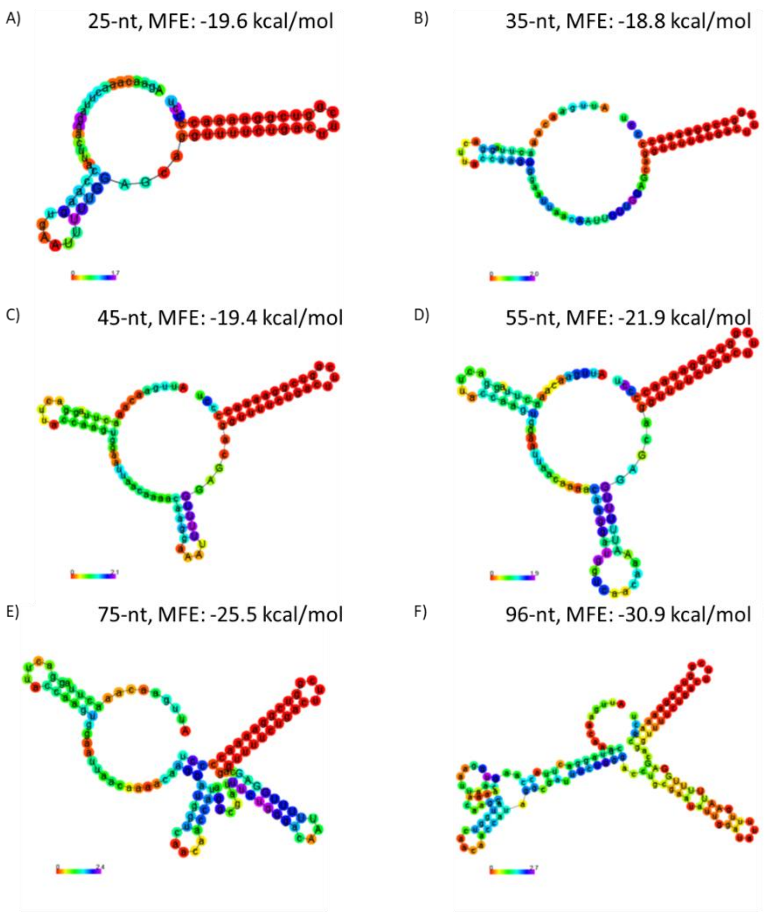

Supplement: S2 Fig — Reliability plot and minimum free energy (MFE) of antisense sequences on modified U7 snRNA targeting mouse Fas exon 6. A) 25-nt, B) 35-nt, C) 45-nt, D) 55-nt, E) 75-nt, and F) 96-nt respectively. ViennaRNA packages version 2.5.1 was used for prediction. (PDF) [file pone.0305012.s007.pdf]
