# Supplementary material for: Design and evaluation of antisense sequence length for modified mouse U7 small nuclear RNA to induce efficient pre-messenger RNA splicing modulation in vitro
Source: PLoS One. 2024 Jul 9;19(7):e0305012. doi: 10.1371/journal.pone.0305012 (PMC11232981; doi:10.1371/journal.pone.0305012)

S4 Fig. Prediction of secondary structure formed by long antisense sequences on modified U7 snRNA.

Reliability plot and minimum free energy (MFE) of antisense sequences on modified U7 snRNA targeting mouse *Dmd* exon 58. A) 44-nt, B) 54-nt, C) 74-nt, D) 94-nt, and E) 114-nt, respectively. ViennaRNA packages version 2.5.1 was used for prediction.

S4 Fig.

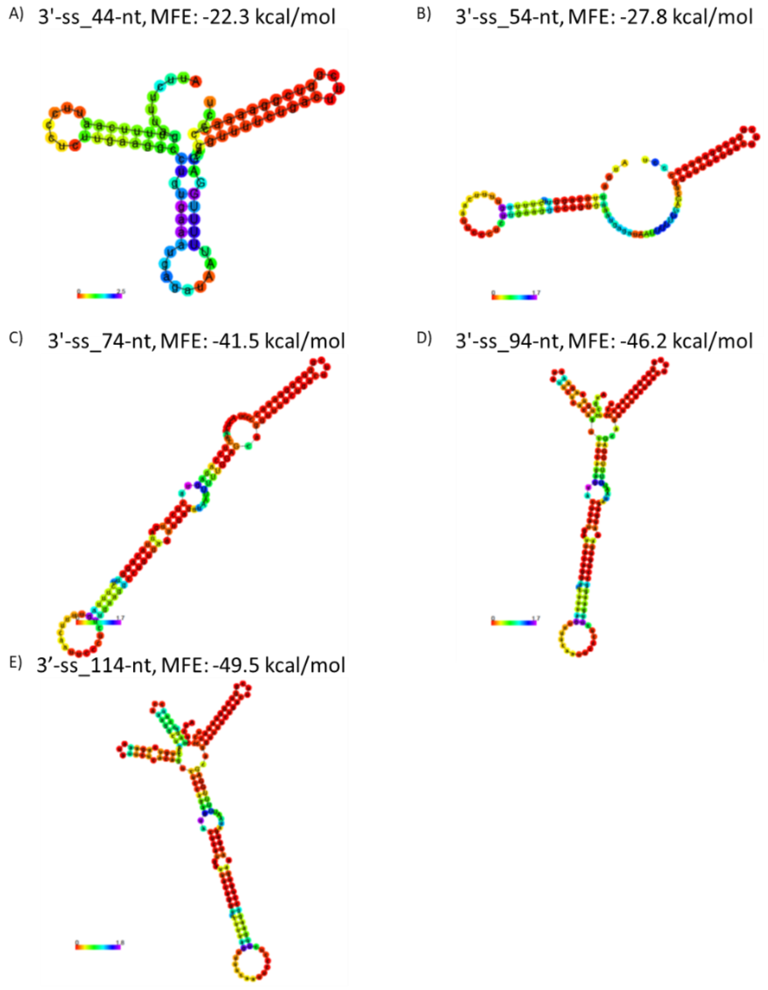

Supplement: S4 Fig — Reliability plot and minimum free energy (MFE) of antisense sequences on modified U7 snRNA targeting mouse Dmd exon 58. A) 44-nt, B) 54-nt, C) 74-nt, D) 94-nt and E) 114-nt respectively. ViennaRNA packages version 2.5.1 was used for prediction. (PDF) [file pone.0305012.s009.pdf]
