# Supplementary material for: Design and evaluation of antisense sequence length for modified mouse U7 small nuclear RNA to induce efficient pre-messenger RNA splicing modulation in vitro
Source: PLoS One. 2024 Jul 9;19(7):e0305012. doi: 10.1371/journal.pone.0305012 (PMC11232981; doi:10.1371/journal.pone.0305012)

S1 raw images

A)

The original gel for Fig 2. This gel indicates the results of the RT-PCR analysis targeting the mouse Fas gene.

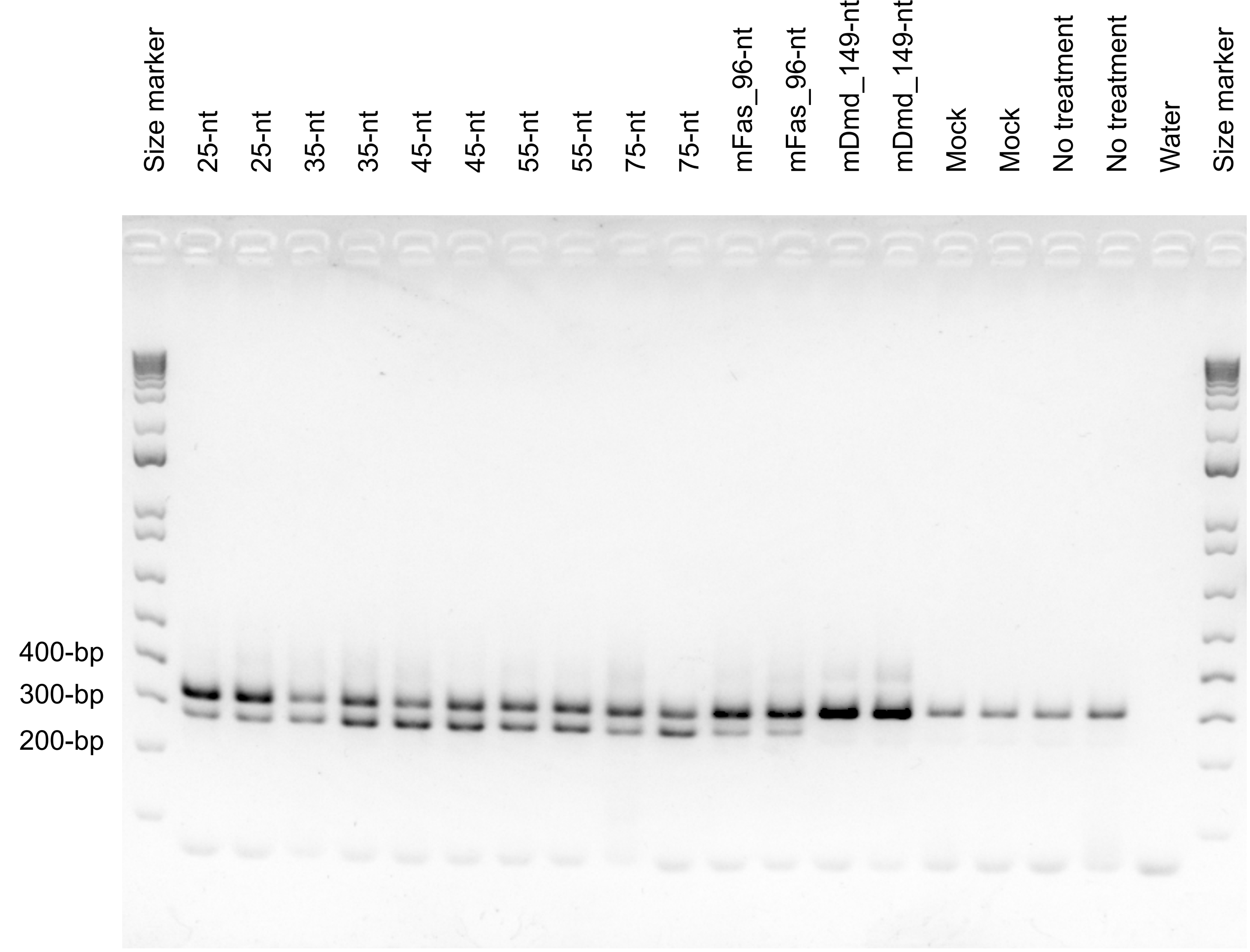

B)

The original gel for Fig 2. This gel indicates the results of the RT-PCR analysis targeting the mouse Actb gene.

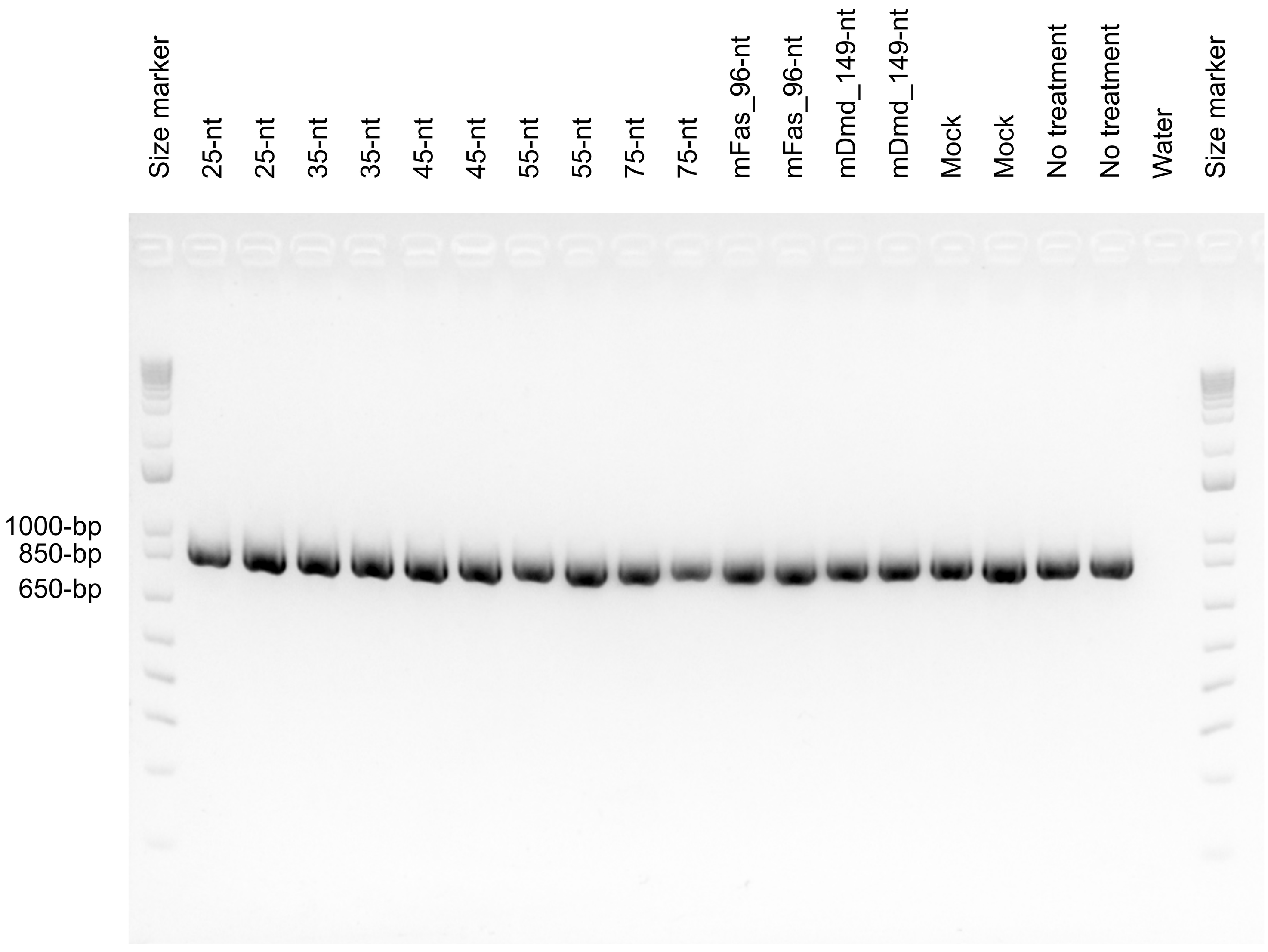

S1 raw images (continued)

C)

The original gel for Fig 3. This gel indicates the results of the RT-PCR analysis targeting the mouse Dmd minigene.

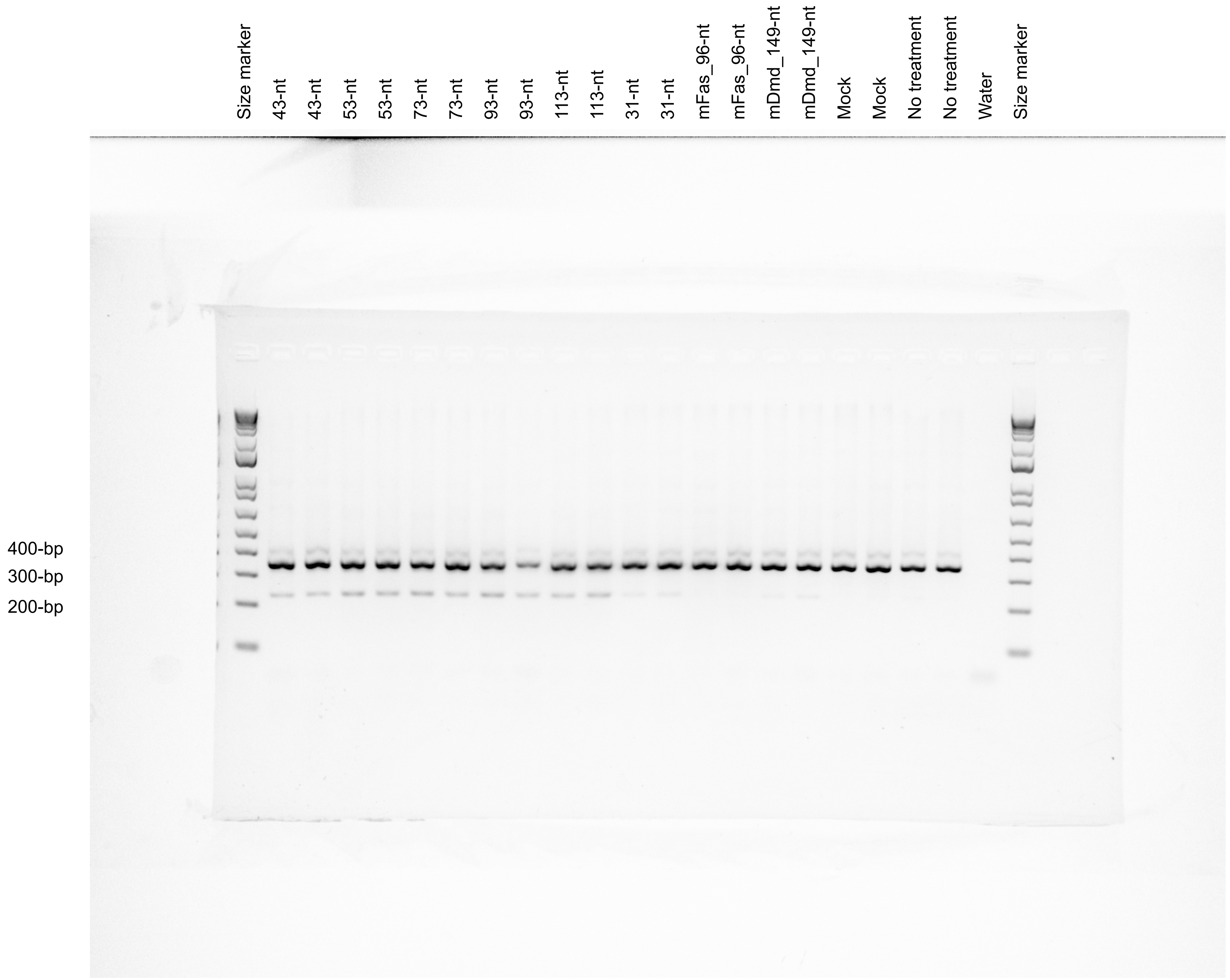

D)

The original gel for Fig 3. This gel indicates the results of the RT-PCR analysis targeting the mouse Actb gene.

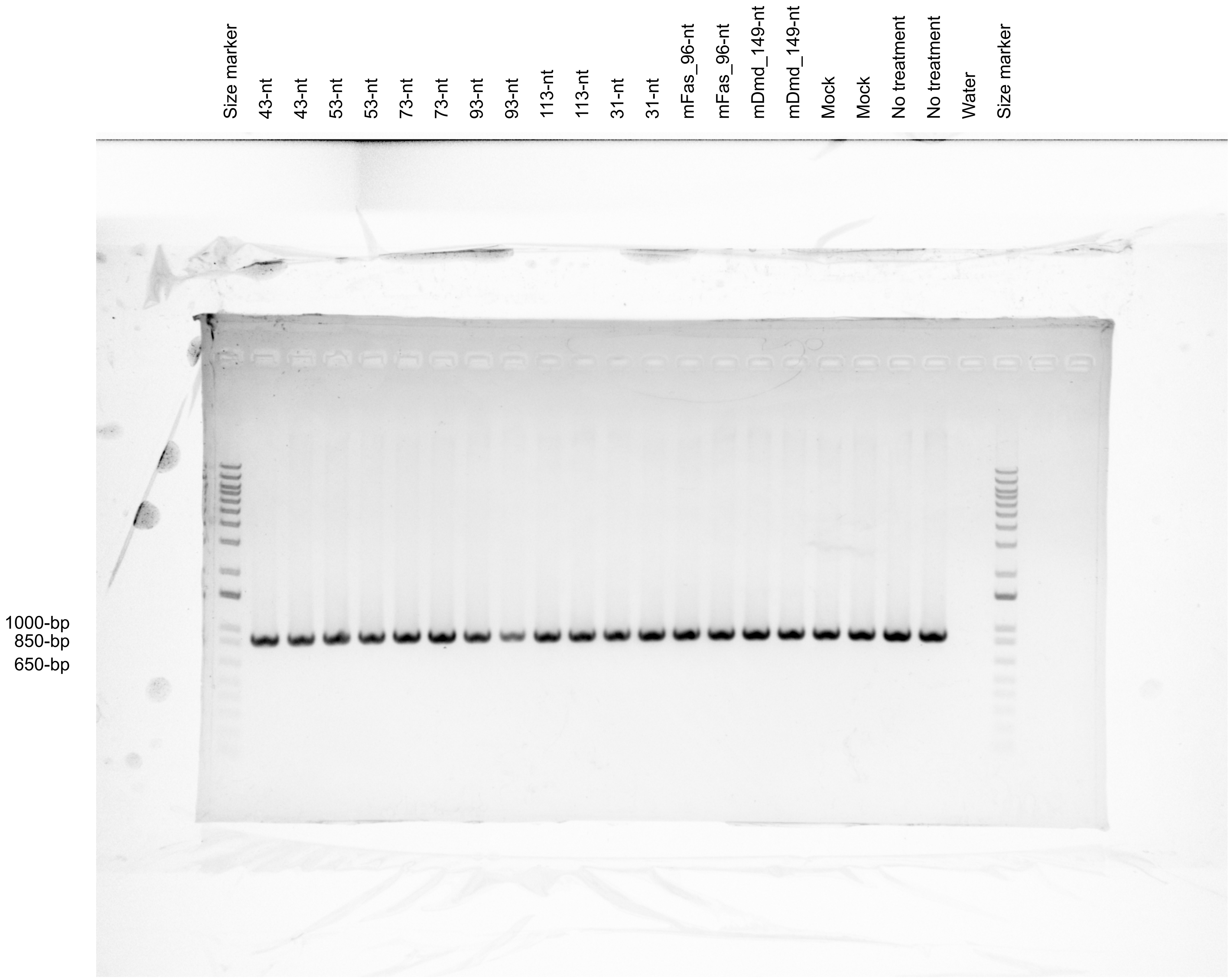

S1 raw images (continued)

E)

The original gel for Fig 4. This gel indicates the results of the RT-PCR analysis targeting the mouse Dmd minigene.

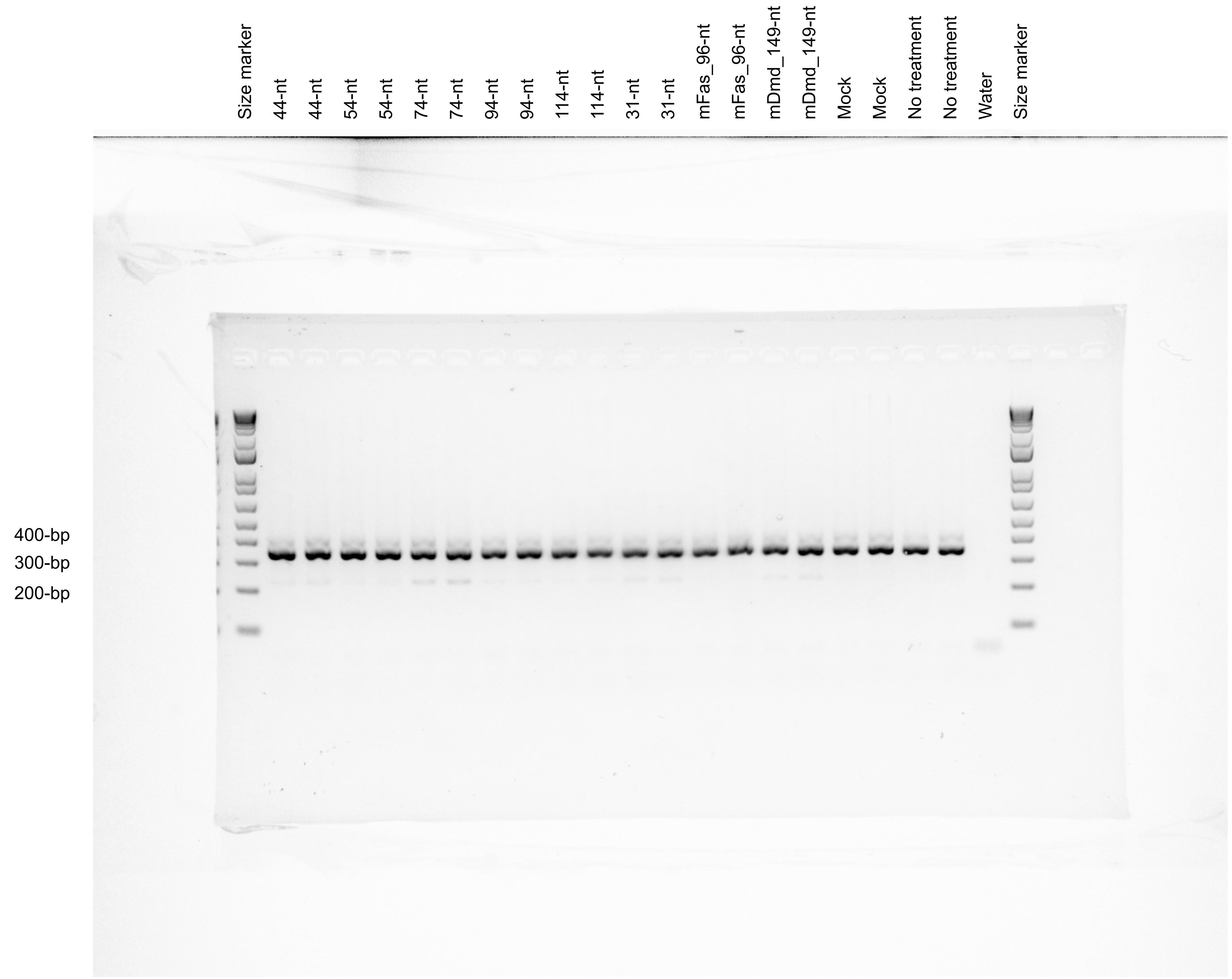

F)

The original gel for Fig 4. This gel indicates the results of the RT-PCR analysis targeting the mouse Actb gene.

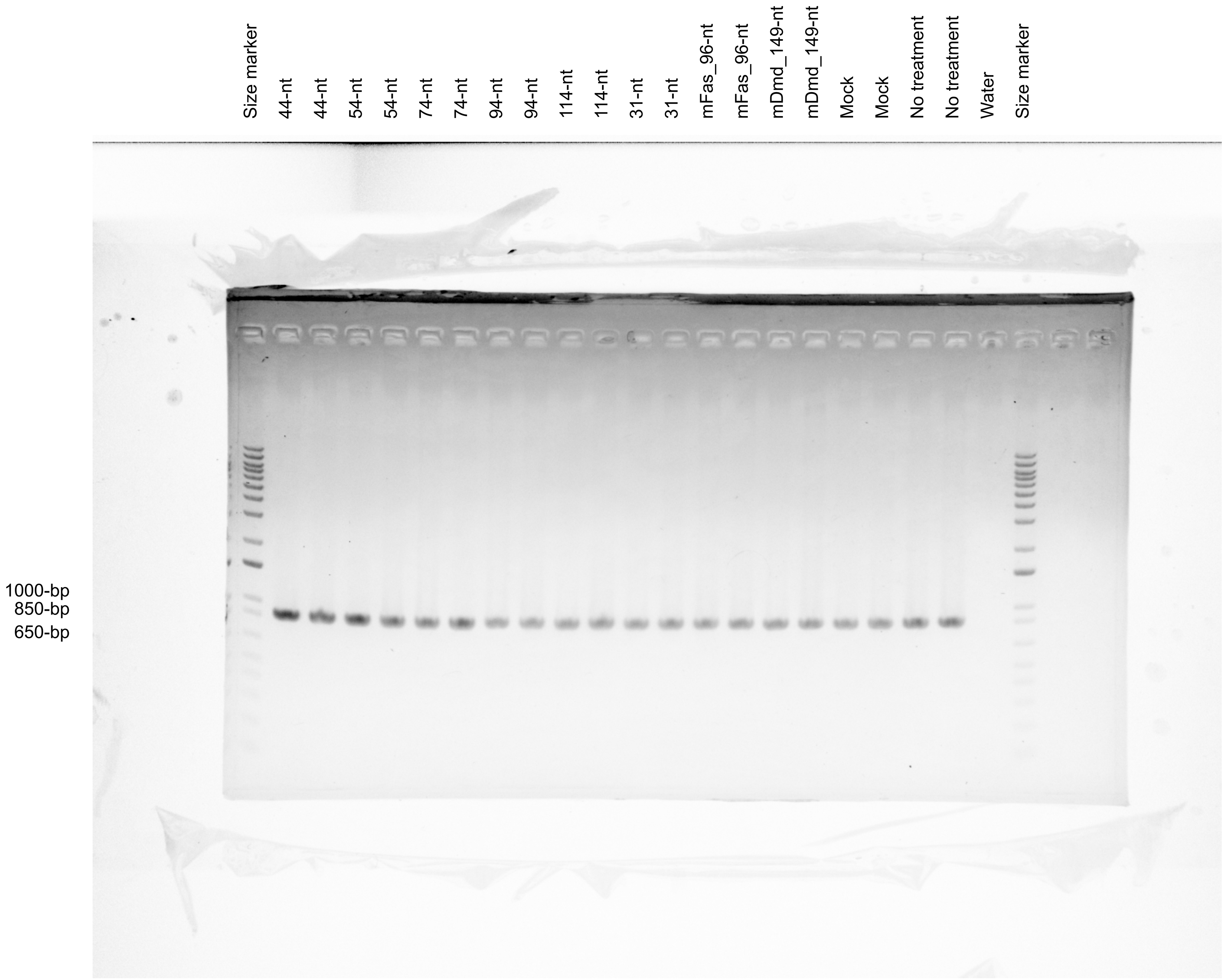

Supplement: S1 Raw image — A) The original gel for Fig 2. This gel indicated the result of RT-PCR analysis targeting mouse Fas. B) The original gel for Fig 2. This gel indicated the result of RT-PCR analysis targeting mouse Actb. C) The original gel for Fig 3. This gel indicated the result of RT-PCR analysis targeting mouse Dmd minigene. D) The original gel for Fig 3. This gel indicated the result of RT-PCR analysis targeting mouse Actb. E) The original gel for Fig 4. This gel indicated the result of RT-PCR analysis targeting mouse Dmd minigene. F) The original gel for Fig 4. This gel indicated the result of RT-PCR analysis targeting mouse Actb. (PDF) [file pone.0305012.s010.pdf]
